# Impact of hookworm infection and deworming on anaemia in non-pregnant populations: a systematic review

**DOI:** 10.1111/j.1365-3156.2010.02542.x

**Published:** 2010-07

**Authors:** Jennifer L Smith, Simon Brooker

**Affiliations:** 1 Department of Infectious and Tropical Diseases, London School of Hygiene and Tropical Medicine, UK; 2 Malaria Public Health and Epidemiology Group, Centre for Geographic Medicine, Kenya Medical Research Institute-Wellcome Trust Research Programme, Nairobi, Kenya

**Keywords:** hookworm, *Necator americanus*, *Ancylostoma duodenale*, anaemia, haemoglobin, anthelmintic treatment

## Abstract

**Objectives:**

To summarise age- and intensity-stratified associations between human hookworm infection and anaemia and to quantify the impact of treatment with the benzimidazoles, albendazole and mebendazole, on haemoglobin and anaemia in non-pregnant populations.

**Methods:**

Electronic databases (MEDLINE, EMBASE, PubMed) were searched for relevant studies published between 1980 and 2009, regardless of language, and researchers contacted about potential data. Haemoglobin concentration (Hb) was compared between uninfected individuals and individuals harbouring hookworm infections of different intensities, expressed as standardised mean differences (SMD) and 95% confidence intervals (CI). Meta-analysis of randomised control trials (RCTs) investigated the impact of treatment on Hb and anaemia.

**Results:**

Twenty-three cross-sectional studies, six pre- and post-intervention studies and 14 trials were included. Among cross-sectional studies, moderate- and heavy-intensity hookworm infections were associated with lower Hb in school-aged children, while all levels of infection intensity were associated with lower Hb in adults. Among RCTs using albendazole, impact of treatment corresponded to a 1.89 g/l increase (95%CI: 0.13–3.63) in mean Hb while mebendazole had no impact. There was a positive impact of 2.37 g/l (95%CI: 1.33–3.50) on mean Hb when albendazole was co-administered with praziquantel, but no apparent additional benefit of treatment with benzimidazoles combined with iron supplementation. The mean impact of treatment with benzimidazoles alone on moderate anaemia was small (relative risk (RR) 0.87) with a larger effect when combined with praziquantel (RR 0.61).

**Conclusions:**

Anaemia is most strongly associated with moderate and heavy hookworm infection. The impact of anthelmintic treatment is greatest when albendazole is co-administered with praziquantel.

## Introduction

Hookworms (*Necator americanus* and *Ancylostoma duodenale*) reside in the small intestine of infected individuals where they attach themselves to the villi and feed on host blood. Among individuals with inadequate iron intake and high physiological demands, this blood loss can result in anaemia. The link between hookworm and anaemia was first established in the nineteenth century (Perroncito 1880), and during the subsequent 130 years, there have been numerous reviews of the extensive literature in this area (Layrisse & Roche 1964; Miller 1979; Schad & Banwell 1984; Crompton & Stephenson 1990; Crompton & Whitehead 1993; Stoltzfus *et al.* 1997; Brooker *et al.* 2004; Hotez *et al.* 2004). There is a direct relationship between the number of hookworms an individual harbours (the intensity of infection) and the amount of intestinal blood lost attributable to hookworm (Gilles & Williams 1964; Martinez-Torres *et al.* 1967; Stoltzfus *et al.* 1996). The clinical consequences of this loss will depend on the host’s underlying iron status as well as the presence of other causes of anaemia (Fleming 2000). Studies indicate that there is some worm burden threshold above which clinically significant anaemia is likely to occur, with the precise threshold dependent on the host’s iron status (Lwambo *et al.* 1992). As well as influencing morbidity, worm burden is a key determinant of transmission dynamics and hence the rate of reinfection following anthelmintic treatment (Anderson & May 1985). Intensity of infection may also influence the efficacy of treatment (Bennett & Guyatt 2000). It follows therefore that as the intensity of hookworm infection varies considerably between populations, the risk of anaemia attributable to hookworm and the impact of treatment will differ among populations.

In 2007, a systematic review of randomised controlled trials (RCTs) investigating the impact of anthelmintic treatment reported an increase in haemoglobin concentration (Hb) of 1.71 g/l after treatment (Gulani *et al.* 2007). But this review did not distinguish between different helminth species or account for intensity of infection, which may have underestimated the true treatment effect (Awasthi & Bundy 2007); the effect of treatment is likely to be greatest where hookworm is most prevalent and intense. Recent work has quantified hookworm-related anaemia among pregnant women (Brooker *et al.* 2008). The present work aims to quantify the impact of hookworm infection and anthelmintic treatment using benzimidazoles, albendazole and mebendazole, among non-pregnant populations in hookworm-endemic areas. Specifically, we review available data from cross-sectional studies that investigated the relationship between intensity of hookworm infection and Hb. We also summarise available data from RCTs and pre- and post-intervention observational studies that compared the effects of benzimidazole treatment, either alone or in combination with the anti-schistosomal drug praziquantel, on Hb and anaemia levels. Finally, based on the value of combining deworming with micronutrient supplementation in children, we evaluate the impact of treatment in combination with iron supplementation (Hall 2007).This work contributes to the current reassessment of the global burden of disease (Murray *et al.* 2007).

## Methods

### Identification of cross-sectional studies

The bibliographic databases of MEDLINE (http://medline.cos.com/), EMBASE (http://www.embase.com/) and PubMed (http://www.ncbi.nlm.nih.gov/pubmed/) were searched for relevant studies in 2006 and again in April 2009. For analysis of the association between intensity of hookworm infection and anaemia, the following Medical Subject Headings (MSHs) were used to identify relevant studies published between 1980 and 2009: *hookworm*, *Necator americanus*, *Ancylostoma duodenale*, *an(a)emia*, *h(a)emoglobin* and *h(a)ematocrit*. Cross-sectional studies published prior to 1980 were reviewed, but those presenting relevant statistics were found to use different diagnostic test and intensity thresholds, making comparisons with later studies difficult (Beaver 1951; Carr 1926; Chernin 1954). Returned abstracts were reviewed and full texts retrieved if they contained relevant information. References from articles and key reviews were screened for additional studies. Finally, leading researchers in the area and authors of key papers were contacted to ask about unpublished or unindexed data, and this yielded a number of additional studies. Non-English language journals were included in the search, and relevant articles were assessed against the inclusion/exclusion criteria by native speakers. No distinction could be made between the two different hookworm species, *Necator americanus* and *Ancylostoma duodenale,* as none of the studies used diagnostic methods able to differentiate species.

The primary outcome for analysis was haemoglobin concentration (Hb) in non-pregnant populations, and our hypothesis was that haemoglobin concentration is associated with the intensity of hookworm infection as assessed by quantitative egg counts, expressed as eggs per gram (epg)/faeces. Abstracted data included the mean Hb, corresponding standard deviation (SD) and number of individuals infected for each category of hookworm infection intensity and were entered into an Excel database. When data were not reported in the preferred format, authors were contacted to request relevant data summaries. Data were stratified by age group (0–4, 5–19 and 20+ years) and category of infection intensity (light, 0–1999 epg; moderate, 2000–3999 epg; heavy, 4000+ epg)) (WHO 2002).

### Identification of treatment studies

Treatment studies were identified using the MSHs *deworming, anti-helmint(h)ic, anthelmint(h)ic, anthelminth, mebendazole, praziquantel, pyrantel, piperazine, nitazoxanide, levamisole, albendazole, bephenium* and *niclosamide*. Only trials that randomised individuals to treatment with a benzimidazole (BMZ) anthelmintic drug and a control group, either placebo or standard of care, and conducted in hookworm-endemic areas were included. Only studies from 1980 onwards were identified because mebendazole was only introduced to the market in 1975 and albendazole in 1980 and use of these benzimidazoles in public health interventions post-dates 1980 (Horton 2003). Two additional groups of studies were included: (i) RCTs of BMZ combined with praziquantel (PQZ) treatment for schistosomiasis and (ii) RCTs of BMZ treatment combined with iron supplementation. In addition to RCTs, observational studies of the impact of intervention were reviewed. Studies that did not quantify the baseline prevalence of hookworm infection, were conducted in pregnant populations, or used an anthelmintic other than albendazole (ABZ) or mebendazole (MBZ) were excluded as these drugs are not widely used in large-scale treatment programmes. Trials were assessed by recommended criteria as shown in Table S2, but quality was not summarised using a score and incomplete reporting was not followed up with authors. These decisions were based on reported unreliability of scales in assessing quality and on the possibility of introducing bias (Higgins & Green 2009).

Primary outcomes were change in mean Hb and prevalence of anaemia, based on the hypothesis that Hb will differ between intervention and control group in response to anthelmintic treatment. Abstracted data included the baseline prevalence of hookworm infection and anaemia, and the post-treatment relative risk of hookworm infection, prevalence of anaemia, mean Hb and change in Hb in each group, with corresponding SDs. For studies that did not report the prevalence of anaemia, an approximation was made on the basis of the reported mean and standard deviation Hb. The proportion of individuals with Hb below the age-specific thresholds for mild, moderate and severe anaemia was calculated assuming a normal Hb distribution (Sharman 2000; WHO 2008). For studies that evaluated treatment effect at multiple time points, only data from the longest time interval were included in the analysis.

### Data analysis

The difference in Hb among different intensity categories was expressed as pool estimates of standardised mean difference (SMD) based on a meta-analysis using a DerSimonian and Laird random effects model. All *P* values are from two-tailed tests of significance where alpha is equal to 0.05.

The impact of treatment was assessed using two approaches. First, for RCTs and observational studies, the impact of treatment on the prevalence of anaemia was expressed as a relative risk (RR) and mean impact summarised. Second, for RCTs, a DerSimonian and Laird random effects meta-analysis was conducted to provide pooled estimates of the effect of treatment on Hb, and a metaregression was used to identify sources of variation between studies. The analysis was stratified by co-administration of PQZ in the intervention arm. This design was justified by the lack of an equivalent intervention in the control arm of these trials and their incomparability to those specifically evaluating the impact of BMZ treatment. SMDs were transformed into g/l using a mean of the SDs of included studies. Linear regression analysis was used to summarise relative risk of mild and moderate anaemia and identify potential determinants of anaemia impact. Study characteristics that were investigated in the modelling process included: age category, WHO region, intervention, baseline prevalence and intensity of hookworm infection, mean Hb at baseline, dosage schedule and follow-up period. Sensitivity analysis identified the Stephenson *et al.* (1993) study as responsible for significant variation in the results, and this study was therefore excluded from further analysis on the basis that participants were restricted to those with heavier infections.

Heterogeneity between studies was assessed by an I^2^ test, with values greater than 50% representing significant heterogeneity, and a sensitivity analysis and preliminary metaregression identified potential sources of variation. Results were displayed as forest plots. Publication bias was investigated by the construction of funnel plots and by the Egger and Begg statistical tests. Analysis was performed using the ‘metan’ and related functions in STATA version 10 (College Station, TX).

## Results

### Associations between hookworm intensity and haemoglobin

The search identified 423 citations, from which 117 unique and potentially relevant articles were retrieved. Of these, 48 were determined to be eligible and 14 had suitable cross-sectional data, including 11 surveys among school-aged children and seven among non-pregnant adults^1^. In addition, unpublished data were available for nine studies. Eighteen studies were conducted in Africa, one in South Asia, four in Southeast Asia and one in Latin America. Survey characteristics are described in Table S1. For all populations, prevalence estimates for hookworm infection ranged from 0.3 to 96%, with 12.5% of the surveys having a mean intensity of infection >1000 epg and eight studies having no individuals with infection intensity >2000 epg. Prevalence of anaemia (110 g/l or 120 g/l threshold) ranged between 4.5 and 90%.

**Figure 1:**
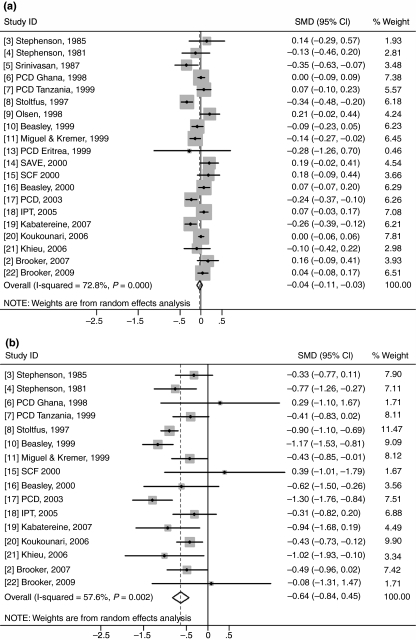
Forest plot of the difference in haemoglobin concentration (Hb) among school-aged children (a) uninfected with hookworm and children with a light (1–1999 eggs/gram) hookworm infection and (b) uninfected with hookworm and children with a heavy (4000+ eggs/gram) hookworm infection. Standardised mean difference less than zero indicates lower Hb levels in children harbouring infections compared to uninfected children. The area of the shaded box represents the contribution (or weight) assigned to the estimate of effect from each study (centre point). The diamond represents the overall pooled estimates of the effect of hookworm infection on Hb. Study ID refer to references in Table S1.

Figure 1 presents the difference in Hb between school-aged children uninfected and those harbouring different levels of infection intensity. There was no evidence for a difference in Hb between uninfected and lightly infected children (SMD −0.04, 95%Confidence interval [CI]: −0.11 to 0.03) (Figure 1a), but there was evidence for a difference between uninfected children and moderately (SMD −0.32, 95% CI −0.46 to −0.18) or heavily (SMD −0.64, 95% CI −0.84 to −0.45) infected children (Figure 1b). There was significant heterogeneity in differences between studies but that could not be explained by any single study. However, a higher baseline prevalence of anaemia was weakly associated with lower Hb in lightly or moderately infected children compared to uninfected children (*P*= 0.06), suggesting that children with poor underlying iron status may be more likely to suffer the consequences of light hookworm infection than those with better nutritional status. There was some evidence of non-symmetry in the funnel plot of uninfected children compared to those with a moderate infection and weak evidence of publication bias using the Egger’s test but not Begg’s test.

**Figure 2:**
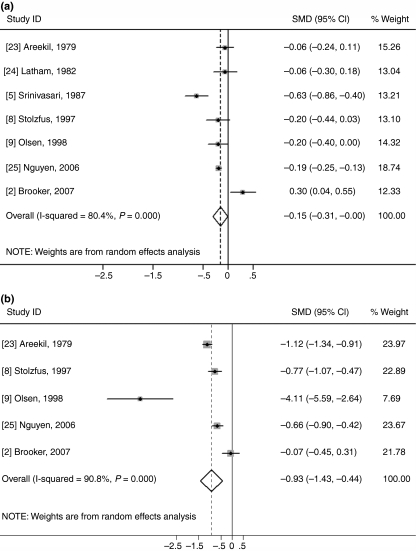
Forest plot of the difference in haemoglobin concentration (Hb) among non-pregnant adults (a) uninfected with hookworm and adults with a light (1–1999 eggs/gram) hookworm infection and (b) uninfected with hookworm and adults with a heavy (4000+ eggs/gram) hookworm infection. Standardised mean difference less than zero indicates lower Hb levels in adults harbouring infections compared to uninfected adults. The area of the shaded box represents the contribution (or weight) assigned to the estimate of effect from each study (centre point). The diamond represents the overall pooled estimates of the effect of hookworm infection on Hb. Study ID refer to references in Table S1.

Among adults, there was evidence for progressively lower Hb among individuals lightly infected (SMD −0.15, 95% CI −0.29 to −0.00) (Figure 2a), moderately infected (SMD −0.47, 95% CI −0.77 to -.17) and heavily infected (SMD −0.93, 95% CI −1.43 to −0.44) relative to those uninfected (Figure 2b). There was evidence of heterogeneity of effect that could be explained by specific studies in each infection strata which, when excluded, altered the SMD (in parenthesis): Brooker *et al.* (2007a), the only study in Latin America (SMD −0.21, 95% CI −0.30 to −0.11); Latham *et al.* (1982) among Kenya male road workers in Kenya (SMD −0.36, 95% CI −0.53 to −0.19); and Olsen *et al.* (1998) in a highly malaria endemic area (SMD −0.71, 95% CI −1.07 to 0.34). No evidence of publication bias was detected in any of the other age group or intensity strata.

### Impact of anthelmintic treatment

Of the 31 studies identified, 14 RCTs met the criteria for inclusion, of which 10 evaluated the effects of either ABZ or MBZ treatment alone (Table 1), four evaluated ABZ treatment with PQZ (Table 2), and five evaluated treatment in combination with iron supplementation (Table 3). In addition, six observational studies were included (Tables 1–3). The majority of studies were conducted in Africa (75%), predominantly in East Africa, used ABZ (85%), and were conducted among school-aged children (75%) (Table 4). Of the RCTs, most studies were individually randomised (79%) and double blind (77%), while seven used a factorial study design to evaluate deworming in combination with iron supplementation. The mean follow-up period for all studies was 3.8 months. Marked variation in the prevalence of hookworm and anaemia (using different thresholds) existed between studies.

**Table 4:** Summary of 20 hookworm intervention studies investigating the impact of benzimidazole treatment (BMZ) on haemoglobin concentration (Hb), administered alone or in combination with praziquantel (PQZ) or iron

		Number of studies	Mean (range)
**Region**	**Sites**		
Asia, South	Bangladesh	1	
	India	1	
Asia, Southeast	Viet Nam	3	
sub-Saharan Africa, East	Kenya	4	
	Tanzania	5	
	Uganda	1	
sub-Saharan Africa, Southern	South Africa	1	
sub-Saharan Africa, West	Benin	1	
	Burkina Faso	1	
	Côte d’Ivoire	1	
	Niger	1	
Study design:	Observational studies	6	
	Randomised controlled trials	14	
Benzimidazole type	Albendazole	17	
	Mebendazole	3	
Assessed	BMZ alone	11	
	BMZ + PQZ	9	
	BMZ + iron	5	
	BMZ + PQZ + iron	3	
Age category (years)	0–4	3	
	5–18	15	
	18–70	2	
Study duration (months)		20	9.4 (1.6–24)
Mean baseline hookworm prevalence (%)		20	50.7 (3.6–100)
Mean baseline hookworm intensity (epg)		14	1387(5.6–6229)
Mean relative risk of infection*		14	0.58 (0–1.2)
***BMZ Alone***			
Comparison arm mean change in Hb†		10	2.5 (−6 to 15.4)
Intervention arm mean change in Hb†		10	4.0 (−4 to 14.6)
Increase in mean change in Hb		11	+2.3 (−0.8 to 9.3)
Mean relative risk of Hb<115 g/l		11	0.91 (0.56–1.5)
Mean relative risk of Hb<100 g/l		10	0.77 (0.21–1.34)
***BMZ + PQZ‡***			
Comparison arm mean change in Hb†		4	−1.9 (−5.8 to 5.5)
Intervention arm mean change in Hb†		4	0.6 (−3.8 to 8.6)
Increase in mean change in Hb		8	+3.7 (2–8.8)
Mean relative risk of Hb<115 g/l		8	0.72 (0.31–0.9)
Mean relative risk of Hb<100 g/l		7	0.58 (0.23–0.8)
***BMZ + iron‡***			
Comparison arm mean change in Hb†		4	9.2 (2.4–17.8)
Intervention arm mean change in Hb†		4	10.2 (2.5–17.5)
Increase in mean change in Hb		5	+2.7 (−0.3–9.2)
Mean relative risk of Hb<115 g/l		5	0.93 (0.51–1.2)
Mean relative risk of Hb<100 g/l		5	0.67 (0.1–1.6)
***BMZ + PQZ + iron‡***			
Comparison arm mean change in Hb†		3	1.0 (−3.9 to 9.2)
Intervention arm mean change in Hb†		3	4.0 (−1.9 to 10.4)
Increase in mean change in Hb		2	+3.0 (1.2–5.9)
Mean relative risk of Hb<115 g/l		2	0.94 (0.9–1.0)
Mean relative risk of Hb<100 g/l		2	1.11 (0.9–1.3)

* The reduction in the prevalence of hookworm infection at the time of follow-up (intervention group/control group).

† Limited to randomised controlled trials.

‡ PQZ administered in the intervention arm only, iron in both intervention and control groups.

**Table 3:** Anthelmintic intervention studies investigating the impact of albendazole (ABZ) or mebendazole (MBZ) combined with iron supplementation on anaemia outcomes in non-pregnant populations

											Anaemia prevalence, mean Hb ± SD or change in Hb (SE)§			
Site & year	Intervention*	Age (years)	Study duration (months)	Parasite prevalence	Hw mean intensity (epg)	RR of Hw infection†	Prevalence of anaemia & mean Hb ± SD	Outcome measure	Hb impact‡	Anaemia impact: RR	Intervention	Control	*N*¶	Reported data or normal approx.**
Randomised-controlled trials (combined with iron; iron + AH placebo in control group)														
Benin, (Dossa *et al.* 2001)	ABZ (200 mg) at 0, 1 months. Iron (60 mg/day)	3–5	10	Hw= 13 Al= 38 Tt= 47	755‡‡‡	0.23 (3 months)	76% (<100 g/l) 100.5 ± 11	Mean Change <110 g/l <100 g/l	+2 +2	0.89 1.17	113 ± 13 +13 (2.6) 40.9% 15.9%	111 ± 10 +11 (2.1) 46.0% 13.6%	34	Data Data Approximation Approximation
Bangladesh, (Gilgen 2001)	ABZ (400 mg) single dose at 0, 12 weeks. Ferrous fumarate (200 mg) + folic acid (200 mg)	14–66††	5.5	Hw= 74.4 Al= 47.6 Tt= 56.8	57.7‡‡‡	NR	86% (<120 g/l) 99.3	Mean Change‡‡^,^§§	+1.9 +2.3		106.9 +7.8 (1.3)	105.0 +5.5 (1.3)	130	Data Data
Zanizibar, (Stoltzfus *et al.* 2004)***	MBZ (500 mg) single dose every 3 months Ferrous sulfate (10 mg)	0.5–5	12	Hw= 31.3 Al= 31.3 Tt= 47.7	5.6‡‡‡	0.74	94% (<110 g/l) 91 ± 12	Mean Change <110 g/l: <30 months ≥30 months <90 g/l: <30 months ≥30 months. <70 g/l:	+1.0 +1.0	1.2 1.0 0.71 1.59 1.06	100±16 +9 80.0% 81.3% 26.0% 17.2% 1.8%	99±16 +8 65.9% 79.7% 36.4% 10.8% 1.7%	22022050 64 50 64 114	Combined data Combined data Data Data Data Data Data
Viet Nam, 2004 (Le Huong *et al.* 2007)	MBZ (500 mg) single dose at 0, 3 months Iron fortified noodles (10.7 mg/52 g noodle)	6–8	6	Hw= 8.5 Al= 67.9 Tt= 77.6	Mostly ‘light’	0.28	87% (<115 g/l) 107.4 ± 7.6	Mean¶¶ Change <115 g/l <100 g/l	−0.5 −0.3	1.09 0.12	124.8 ± 6.8 +17.5(0.85) 11.4% 0%	125.3 ± 8.3 +17.8 (0.97) 10.5% 0.1%	79	Data Data Data Approximation
Viet Nam, 2007 (Nga *et al.* 2009)	ABZ (400 mg) single dose Micronutrients	6–8	4	Hw= 6.2 Al= 64.8 Tt= 22.8	Mostly ‘light’	1.2	26% (<115 g/l) 119.3 ± 7.5	Mean Change‡‡ <115 g/l <100 g/l	+1.0 +0.1	0.86 0.09	122.2 ± 6.2 +2.5 12.8% 0%	121.2 ± 7.3 +2.4 14.9% 0.2%	118	Data Data Data Approximation
Randomised-controlled trials (combined with praziquantel (PQZ) & iron; iron + AH placebo in control group)														
South Africa, 1996 (Taylor 2001)***	ABZ(400 mg) triple dose at 0, 6 months PQZ Ferrous fumarate 200 mg/wk × 10	6–15	12	Hw= 59.4 Pf= 5.1 Sm= 0	Light	NR	34% (<120 g/l) 122.7	Mean¶¶ Change	+3.5 +5.9		124.7 +3.5 (1.5)	121.2 −2.4 (1.2)	41	Data Data
Kenya, 2003 (Friis 2003)	ABZ(600 mg) single dose PQZ Micronutrients	8–18	8	Hw= 58.2 Al= 13.3 Tt= 42.7 Pf= 58.7 Sm= 69.6	45‡‡‡	NR	40% (age/sex specific) 123.6 ± 12.1	Mean¶¶ Change <115 g/l <100 g/l	+1.6 +1.2	0.99 1.34	134.2 ± 12.6 +10.4 (1.1) 6.4% 0.3%	132.6 ± 11.6 +9.2 (1.0) 6.5% 0.2%	180	Data Data Approximation Approximation
Côte d’Ivoire, 2007 (Rohner *et al.* 2010)	ABZ (400 mg) single dose at 0, 3 months PQZ Fortified biscuits (20 mg Fe 4 times/week)	6–14	6	Hw= 53.4 Al= 1.4 Tt= 2.9 Pf= 56.1	107.8‡‡‡	0.27	72% (<115 or 120 g/l) 111.2 ± 10.6	Mean Change <115 g/l <100 g/	+2.1 +2.0	0.88 0.89	109.3 ± 10.7 −1.9 (0.7) 78.7% 19.2%	107.2 ± 9.2 −3.9 (1.3) 89.0% 21.7%	64	Data Data Data Approximation.
Observational study											Baseline	Follow-up		
Viet Nam, 2005 (Casey *et al.* 2009)	ABZ (400 mg) single dose every 4 months Ferrous sulphate/folic acid (60 mg/0.4 mg)	15–45††	12	Hw= 76.2	NR	0.38	38% (<120 g/l) 122.5†††	Mean¶¶ <120 g/l <100 g/l	+9.2	0.51 0.35	132.0 19.3% 3.0%†††	122.5 37.5% 8.5%†††	382	Data Data Data

Hw, hookworm; Al, *Ascaris lumbricoides*; Tt, *Trichuris trichiura*; SD, standard deviation; SE, standard error; NR, not reported; RR, relative risk.

* PQZ administered by WHO praziquantel dose pole (40 mg/kg).

† The reduction in the prevalence of hookworm infection at the time of follow-up (intervention group/control group).

‡ The difference in mean Hb or mean change in Hb between intervention and control groups at follow-up.

§ Mean Hb in g/l.

¶ Number in treatment group at follow-up.

** Approximation of the prevalence of anaemia, assuming Hb concentrations to be normally distributed around the reported mean, with the reported SD.

†† Gilgen *et al.* 2001 and Casey *et al.* 2009 only include women.

‡‡ Change in Hb was estimated as the difference in reported pre-intervention and post-intervention Hb levels.

§§ Mean Hb is estimated from baseline Hb and change in Hb and assumed to have the same SD as at baseline.

¶¶ Values estimated from graph.

*** The adjusted odds ratio (age, baseline Hb, fever, *Plasmodium falciparum*) followed the same trend as crude RR.

††† Standard error estimated from t-test.

‡‡‡ Geometric mean.

**Table 2:** Anthelmintic intervention studies investigating the impact of albendazole (ABZ) or mebendazole (MBZ) combined with praziquantel (PQZ) on anaemia outcomes in non-pregnant populations

											Anaemia prevalence, mean Hb ± SD or change in Hb (SE)§			
Site & year	Intervention*	Age (years)	Study duration (months)	Parasite prevalence	Hw mean intensity (epg)	RR of Hw infection†	Prevalence of anaemia & mean Hb ± SD	Outcome measure	Hb impact‡	Anaemia impact	Intervention	Control	*N*¶	Reported data or normal approx.**
Randomised-controlled trials (combined with praziquantel (PQZ); placebo in control group)														
Tanzania, 1994 (Beasley *et al.* 1999)	ABZ (400 mg) single dose PQZ	7–12	3.5	Al= 49.1 Hw= 92.9 Tt= 68.2 Pf= 74.1 Sh= 100	2045	0.63	49% (<110 g/l) 110 ± 10.1	Mean Change <110 g/l <100 g/l	+2.0 +2.4	0.85 0.60	109 ± 9.0 −1.1 (.7) 53% 15.9%	107 ± 11.1 −3.5 (.7) 62% 26.4%	127	Data Data Data Approximation
South Africa, 1996 (Taylor *et al.* 2001)	ABZ (400 mg) triple dose at 0, 6 months PQZ	6–15	12	Hw= 59.4 Pf= 5.1 Sm= 0	Light	NR	34% (<120 g/l) 125.3	Mean†† Change	+2.1 +2.0		121.6 −3.8 (1.6)	119.5 −5.8 (1.2)	34	Data Data
Kenya, 2003 (Friis *et al.* 2003)	ABZ (600 mg) single dose PQZ	9–18	8	Hw= 51.9 Al= 14.0 Tt= 48.1 Pf= 59.5 Sm= 72.8	59‡‡	NR	40% (age/sex specific) 123.7 ± 12.7	Mean†† Change <115 g/l <100 g/l	+3.9 +3.1	0.31 0.62	132.7 ± 12.7 +8.6 (0.9) 4.0% 0.6%	128.8 ± 12.3 +5.5 (1.1) 13.1% 1.0%	187	Data Data Approximation Approximation
Côte d’Ivoire, 2007 (Rohner *et al.* 2010)	ABZ (400 mg) single dose at 0, 3 months PQZ	6–14	6	Hw= 51.4 Al= 1.4 Tt= 2.9 Pf= 57.7	107.8‡‡	0.38	70% (<115 or 120 g/l) 110.8 ± 9.0	Mean Change <115 g/l <100 g/	+2.9 +2.7	0.80 0.62	109.6 ± 9.2 −1.2 (1.0) 70.6% 14.8%	106.7 ± 9.4 −3.9 (1.1) 87.8% 23.8%	60	Data Data Data Approximation
Observational studies											Baseline	Follow-up		
Tanzania, 1996 (Guyatt *et al.* 2001)	ABZ (400 mg) single dose PQZ	8–14	10, 15	Hw= 61 Sh= 59	738	0.80	54% (<110 g/l) 10% (<90 g/l) 107.3 ± 14.5	Mean <110 g/l <90 g/l <70 g/l	+5.5	0.74 0.63 0.53	112.8 ± 15.1 40.0% 6.1% 0.8%	107.3 ± 14.5 54.1% 9.7% 1.5%	1121	Data Data Data Data
Tanzania, 1997 (Bhargava *et al.* 2003)	ABZ (400 mg) triple dose at 0, 12 weeks PQZ	SAC	15	Hw= 100 Sh= 100	423	NR	67% (<120 g/l) 112.1 ± 15.2	Mean <115 g/l <100 g/l	+8.8	0.34 0.23	120.9 ± 11.4 19.3% 4.9%	112.1 ± 15.2 57.6% 21.3%	135	Data Data Approximation Approximation Approximation
Uganda, 2003 (Koukounari *et al.* 2006)	ABZ (400 mg) single dose PQZ	6–14	12	Hw= 52.1 Al= 2.4 Tt= 2.3 Sm= 43.9	307	0.46	50% (<115 or 120 g/l) 114.3 ± 13.5	Mean <115 g/l <100 g/l <70 g/l	+2.4	0.92 0.75 0.62	116.7 ± 13.5 45.8% 10.8% 0.18%	114.3 ± 13.5 50.0% 14.5% 0.29%	2788	Data Data Approximation Data
Burkina Faso, 2004 (Koukounari *et al.* 2007)	ABZ (400 mg) single dose PQZ	5–15	12	Hw= 6.3 Sm= 6.2 Sh= 53.9	12.5	0.68	66% (<115 or 120 g/l) 110 ± 14	Mean <115 g/l <100 g/l	+2.8	0.94 0.61	112.5 ± 12 61.6% 14.9%	109.7 ± 14 65.8% 24.4%	1131	Data Data Approximation
Niger, 2004 (Tohon *et al.* 2008)	ABZ (400 mg) single dose PQZ	7, 8, 11	12	Hw= 4.2 Al= 0.3 Tt= 0.09 Sm= 0.9 Sh= 75.4 Pf= 8	NR	NR	62% (<115 g/l) 110	Mean <115 g/l	+4	0.81	114 g/l 50.4%	110 g/l 61.9%	1642	Data Data

Hw, hookworm; Al, *Ascaris lumbricoides*; Tt, *Trichuris trichiura*; SD, standard deviation; SE, standard error; NR, not reported; RR, relative risk.

* PQZ administered by WHO praziquantel dose pole (40 mg/kg).

† The reduction in the prevalence of hookworm infection at the time of follow-up (intervention group/control group).

‡ The difference in mean Hb or mean change in Hb between intervention and control groups at follow-up.

§ Mean Hb in g/l.

¶ Number in treatment group at follow-up.

** Approximation of the prevalence of anaemia, assuming Hb concentrations to be normally distributed around the reported mean, with the reported SD.

†† Mean Hb is estimated from baseline Hb and change in Hb and assumed to have the same SD as at baseline.

‡‡ Geometric mean.

**Table 1:** Anthelmintic intervention studies investigating the impact of albendazole (ABZ) or mebendazole (MBZ) on anaemia outcomes in non-pregnant populations

											Anaemia prevalence, mean Hb ± SD or change in Hb (SE)‡			
Site & year	Intervention	Age (years)	Study duration (months)	Parasite prevalence	Hw mean intensity (epg)	RR of Hw infection*	Prevalence of anaemia & mean Hb ± SD	Outcome measure	Hb impact†	Anaemia impact: RR	Intervention	Control	*N*§	Reported data or normal approx.¶
Randomised-controlled trials														
Kenya (Stephenson *et al.* 1990)	ABZ (400 mg) single dose	6–12 **	1.6	Hw= 91.0 Al= 39.0 Tt= 94.0	6229	0.5	67% (<120 g/l) 113 ± 12	Mean†† Change <115 g/l <100 g/l <70 g/l	0.0 +2.0	0.88 0.62 0.12	110 ± 11.9 −4.0 (1.8) 66.3% 20.0% 0.0%	106 ± 13.2 −6.0 (1.8) 75.2% 32.5% 0.3%	18	Data Data Approximation Approximation Approximation
Kenya, 1989 (Stephenson *et al.* 1993)	ABZ (600 mg) single dose	7–13 **	4	Hw= 96.2 Al= 41.5 Tt= 98.1	3352	0.44	47% (<120 g/l) 120 ± 9.6	Mean Change <115 g/l <100 g/l	+5.0 +4.0	0.65 0.40	119 ± 10.4 −2.0 (1.2) 35.0% 3.4%	114 ± 10.2 −6.0 (1.0) 53.9% 8.5%	27	Data Data Approximation Approximation
Kenya, 1990 (Adams *et al.* 1994)	ABZ (400 mg) triple dose	5–10	1.7	Hw= 92.7 Al= 29.1 Tt= 83.6	4873	0.0	109 ± 14	Mean Change <115 g/l <100 g/l <70 g/l	+2.0 +1.0	0.94 0.81 0.46	108 ± 12.2 −1.6 (1.9) 71.7% 25.6% 0.1	106 ± 12.5 −2.6 (1.6) 78.8% 34.5% 0.3	28	Data Data Approximation Approximation Approximation
Tanzania, 1994 (Stoltzfus *et al.* 1998)	MBZ (500 mg) single dose thrice-yearly	6–16	12	Hw= 93.3 Al= 74.2 Tt= 96.1	450‡‡‡	0.75	63% (<110 g/l) 105 ± 12	Mean†† Change <110 g/l <100 g/l	−0.6 +1.4	1.15 1.33	116.7 ± 13 +12.7(1.7) 33.2% 9.9%	117.3 ± 12 +11.3 (1.7) 28.9% 7.5%	970	Data Data (adjusted) Data Approximation
North India, 1995 (Awasthi & Bundy 2000)‡‡	ABZ (600 mg) single dose, every 6 months.	1.5–3.5	24	Hw= 3.6 Al= 11.7	NR	NR	91% (<110 g/l) 95 ± 9	Mean Change§§ <110 g/l <100 g/l	0.0 0.0	1.00 1.00	96.7 ± 6.6 +1.7 96.8 66.6	96.7 ± 6.5 +1.7 96.8 66.6	610	Data Data Approximation Approximation
Benin, (Dossa *et al.* 2001)	ABZ (200 mg) triple dose at 0,1 month.	3–5	10	Hw= 13 Al= 38 Tt= 47	286‡‡‡	0.23 (3 months.)	76% (<110 g/l) 99.8 ± 11	Mean Change <110 g/l <100 g/l	0.0 +4.0	1.06 0.85	106 ± 10 + 8 (2.1) 65.5% 27.4%	106 ± 13 + 5 (2.1) 62.1% 32.2%	38	Data Data Approximation Approximation
Bangladesh (Gilgen *et al.* 2001)	ABZ (400 mg) single dose at 0 and 12 weeks	14–66**	5.5	Hw= 74.4 Al= 47.6 Tt= 56.8	57.7‡‡‡	NR	86% (<120 g/l) 97.8	Mean Change§§^,^¶¶	+3.9 +2.2		100.6 +2.0 (1.6)	96.7 −0.2 (1.4)	143	Data Data
Tanzania (Stolzfus *et al.* 2004)***	MBZ(500 mg) single dose every 3 months	0.5–5	12	Hw= 31.3 Al= 31.3 Tt= 47.7	5.6‡‡‡	0.74	94% (<110 g/l) 91 ± 12	Mean Change <110 g/l: <30 months >30 months <90 g/l: <30 months >30 months <70 g/l:	+1.0 +1.0	0.98 1.01 0.71 1.34 1.09	100±16 +9 71.4% 73.2% 25.7% 15.5% 5.7%	99±16 +8 87.3% 71.2% 36.6% 13.6% 5.0%	220 220 35 71 35 71 106	Combined data Combined data Data Data Data Data Data
Viet Nam, 2005 (Le Huong *et al.* 2007)	MBZ (500 mg) single dose at 0, 3 months	6–8	6	Hw= 9.3 Al= 68.3 Tt= 68.3	Mostly ‘light’	0.68	88% (<115 g/l) 108.1 ± 6.2	Mean†† Change <115 g/l	−0.1 −0.8	0.78	123.1 ± 6.9 +14.6 (1.0) 15.2%	123.2 ± 6.2 +15.4 (0.92) 19.5%	79	Data Data Data
Viet Nam, 2007 (Nga *et al.* 2009)	ABZ (400 mg) single dose	6–8	4	Hw= 5.1 Al= 66.0 Tt= 62.6	Mostly ‘light’	1.2	24% (<115 g/l) 119.6 ± 7.3	Mean Change§§ <115 g/l <100 g/l	−0.2 +1.2	1.05 0.37	119.9 ± 7 +1.0 20.5% 0.2%	120.1 ± 8 −0.2 19.5% 0.6%	117	Data Data Data Approximation
Observational studies											Baseline	Follow-up		
Tanzania, 1997 (Bhargava *et al.* 2003)	ABZ (400 mg) triple dose at 0, 12 weeks	5–18	3,15	Hw= 100	423	NR	67%††† (<120 g/l) 111.2±16.4	Mean <115 g/l <100 g/l	+9.3	0.56 0.21	120.5 ± 12.6 33.1% 5.2%	111.2 ± 16.4 59.2% 24.7%	56	Data Approximation Approximation

Hw, hookworm; Al, *Ascaris lumbricoides*; Tt, *Trichuris trichiura*; SD, standard deviation; SE, standard error; NR, not reported; RR, relative risk.

* The reduction in the prevalence of hookworm infection at the time of follow-up (intervention group/control group).

† The difference in mean Hb or mean change in Hb between intervention and control groups at follow-up.

‡ Mean Hb in g/l.

§ Number in treatment group at follow-up.

¶ Approximation of the prevalence of anaemia, assuming Hb concentrations to be normally distributed around the reported mean, with the reported SD.

** Stephenson *et al.* 1990, 1993 include men only, Gilgen *et al.* 2001 includes women only.

†† Mean Hb is estimated from baseline Hb and change in Hb and assumed to have the same SD as at baseline.

‡‡ At follow-up, some of control group were treated.

§§ Change in Hb was estimated as the difference in reported pre-intervention and post-intervention Hb levels.

¶¶ Standard error estimated from t-test.

*** The adjusted odds ratio (age, baseline Hb, fever, *Plasmodium falciparum*) followed the same trend as crude RR.

††† Includes children infected with either Hw or *Schistosoma haematobium*.

‡‡‡ Geometric mean.

Across all 20 RCTs and observational studies, the mean change in Hb was higher in the treatment arm for all intervention packages than in the control arm: 2.3 g/l higher in the BMZ group; 3.7 g/l higher in the BMZ and PQZ group; 2.7 g/l higher in the BMZ and iron group; and 3.0 g/l higher in the BMZ, PQZ and iron group (Table 4). The effect of BMZ alone on mild and moderate anaemia was small (mean RR of 0.91 and 0.77), whereas the mean RR of BMZ plus PQZ was 0.72 for mild anaemia and 0.58 for moderate anaemia.

**Figure 3:**
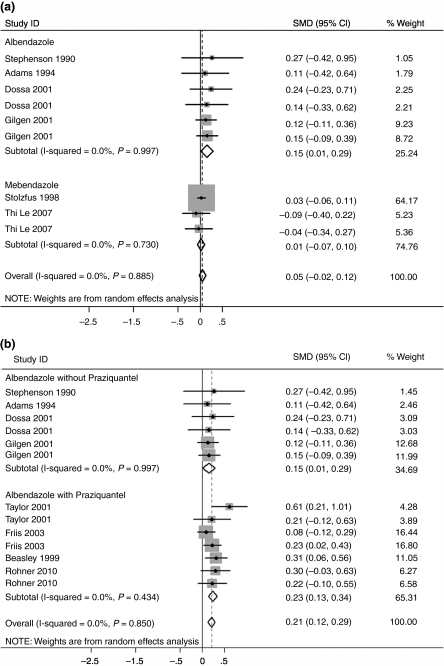
Forest plot of the difference in the mean change in haemoglobin concentration (Hb) among individuals treated with an anthelmintic and individuals given a placebo in interventions studies (*n*= 10). Standardised mean difference greater than zero indicates a greater increase in Hb levels in the treated group (or a smaller decrease) compared to the control group. The area of the shaded box represents the contribution (or weight) assigned to the treatment effect estimated from each study (centre point). Diamonds represent pooled estimates among studies stratified by (a) benzimidazole type in those studies not administering praziquantel and (b) co-administration of praziquantel in the intervention arm. The lowest diamond represents the overall pooled estimates of the effect of any treatment on the mean change in Hb.

Eleven RCTs reported the effect of intervention on mean Hb with corresponding standard deviations (SD) or allowed their estimation (three studies did not report SDs: Awasthi *et al.* 2000; Stoltzfus *et al.* 2004; Nga *et al.* 2009). There was no overall effect of BMZ (SMD 0.05, 95%CI: −0.02 to 0.12), but looking at the drug effects separately, treatment with ABZ corresponded to a 1.89 g/l increase in mean Hb (SMD 0.15, 95% CI 0.01 to 0.29) whereas MBZ had no apparent impact (Figure 3a). Furthermore, combining ABZ and PQZ resulted in a 2.37 g/l increase in mean Hb (SMD 0.23, 95% CI 0.13 to 0.34) (Figures 3b). There was no evidence to support a beneficial impact of BMZ treatment when iron supplementation was co-administered in both arms of the trial (SMD 0.09, 95% CI −0.09 to 0.27) compared to neither arm (SMD 0.04, 95% CI −0.04 to 0.12).

Among RCTs, treatment with BMZ alone had little impact on the risk of mild (RR 0.98, 95%CI: 0.89–1.06) and moderate (RR 0.87, 95%CI, 0.59–1.15) anaemia as determined by linear regression. When BMZ treatment was co-administered with PQZ, the mean relative risks of mild and moderate anaemia were 0.67 (95% CI −0.11 to 1.45) and 0.61 (95% CI 0.58–0.64). Among studies administering BMZ alone, a higher Hb at baseline was associated with a larger impact on moderate anaemia (*P*= 0.02), but there was no evidence of a differential impact when iron supplementation was co-administered in both arms of the trial (*P*= 0.69 and *P*= 0.63). No determinants of impact were identified for studies co-administering BMZ and PQZ.

## Discussion

The aetiology of tropical anaemia is complex, but the present systematic review confirms that hookworm infections of moderate or heavy intensity are associated with lower Hb levels in both school-aged children and adults (Layrisse & Roche 1964; Stoltzfus *et al.* 1997). The mechanisms by which hookworms reduce Hb are well established: adult worms attached to intestinal villi and pass a stream of blood through their intestines to obtain oxygen and nutrients. Fortunately, however, the current results show that anthelmintic treatment is an effective means of improving Hb levels, but that the effects of treatment appear to differ according the benzimidazole drug used (Figure 3). Among the included RCTs, treatment using albendazole was associated with an 1.89 g/l increase in Hb, whereas mebendazole treatment afforded no apparent benefit. However, the impact of benzimidazole treatment on Hb is enhanced by the co-implementation of praziquantel treatment and iron supplementation: for example, the addition of praziquantel resulted in a 2.37 g/l increase.

WHO currently recommends that school-aged children living in areas of high prevalence of soil-transmitted helminths (hookworms, *Ascaris lumbricoides* and *Trichuris trichiura*) receive mass treatment with either albendazole or mebendazole (WHO, 2002). Whilst these drugs are both highly efficacious against *A. lumbricoides*, with demonstrable gains for growth and school performance (Taylor-Robinson *et al.* 2007; Bundy *et al.* 2009), single-dose mebendazole treatment is less effective against *T. trichiura* and hookworm (Keiser & Utzinger 2008). Thus, the differential impact of albendazole and mebendazole on Hb levels can be explained in part by their varying efficacies against hookworm, although it should be noted that observed cure and egg reduction rates of mebendazole against hookworm vary among populations (Keiser & Utzinger 2008). However, this review identified only three published RCTs that investigated the impact of mebendazole on Hb, making it difficult to draw difficult definite conclusions about the effect of mebendazole in different hookworm-endemic regions of the world.

In areas co-endemic with schistosomiasis, benzimidazole is typically co-implemented with praziquantel. Previous attempts to quantify the haematological benefits of praziquantel have been hindered by the lack of RCTs evaluating the effects of PQZ alone (Friedman *et al.* 2005). Praziquantel has a direct effect against schistosomes, which may cause anaemia through a variety of proposed mechanisms, including extra-corporeal blood loss, sequestration of red blood cells, haemolysis and inflammation (Friedman *et al.* 2005). A previous systematic review of schistosomiasis-related morbidity (King *et al.* 2005) identified five studies evaluating the impact of praziquantel on Hb levels in hookworm-endemic areas; but two of these trials co-administered benzimidazole or metrifonate treatment. Metrifonate is partially effective against hookworm infection (Kurz *et al.* 1986), and therefore, inclusion of studies using metrifonate would potentially overestimate the impact of praziquantel treatment. A large multi-centre RCT of praziquantel and albendazole included by King *et al.* (2005) found that only treatment with praziquantel had an impact on Hb, but the estimate of impact for albendazole was not reported (Olds *et al.* 1999).

One of the difficulties of attributing effects of hookworm and anthelmintic treatment on anaemia, particularly among populations exposed to malaria and with inadequate dietary intake, is the exclusion of other causes. The multiple aetiologies of anaemia can confound cross-sectional estimates of association and influence the observed impact of anthelmintic treatment. Malaria (both symptomatic and asymptomatic) is an important aetiological factor for anaemia operating through several mechanisms including increased destruction of red blood cells (RBCs) through rupturing, phagocytosis and hypersplenism and reduced RBC production through inflammation and dyserythropiesis (Kurtzhals *et al.* 1999; Menendez *et al.* 2000; Tolentino *et al.* 2007). Extensive geographic overlap of hookworm and malaria yields a high prevalence of co-infection, which may increase in an additive manner the risk of anaemia (Brooker *et al.* 2007b). A further aetiological factor for anaemia is schistosomiasis, and co-infection with schistosomes and hookworm has been associated with enhanced anaemia risk (Ezeamama *et al.* 2008; Stephenson *et al.* 1985; Brito *et al.* 2006). In the present review, cross-sectional studies did not report adjusted intensity-stratified estimates of Hb and only 5 RCTs stratified results by nutritional status. Studies reported conflicting results: some found a differential impact based on anaemic status (Beasley *et al.* 1999) and intensity of hookworm infection (Stoltzfus *et al.* 1998; Adams *et al.* 1994), while others found no difference in impact between these groups (Taylor *et al.* 2001; Friis *et al.* 2003). Among children with *S. mansoni* infections, Friis *et al.* 2003 reported a greater impact on Hb associated with malaria co-infection, suggesting that malaria may influence the impact of schistosomiasis treatment.

A further potentially confounding factor is anthelmintic treatment efficacy. Reported estimates of the impact of benzimidazole treatment may underestimate the true magnitude of association because of incomplete treatment cure and the potential reinfection that occurs during follow-up (Bradley *et al.* 1993; Stoltzfus *et al.* 2000). Reinfection dynamics of helminths are well described and depend on a number of factors that vary between populations, including transmission intensity, efficacy of treatment and treatment coverage (Anderson & Schad 1985). Studies of hookworm reinfection support the view that prevalence and intensity of hookworm infection can return to pre-treatment levels within 1–2 years, with reinfection fastest in areas of high transmission and where treatment efficacy and coverage is lowest (Quinnell *et al.* 1993; Schad & Anderson 1985; Reynoldson *et al.* 1997 & De Clerq *et al.* 1997). A related issue is variation in follow-up time of included studies because a longer follow-up will allow more opportunities for reinfection and may therefore underestimate the impact of treatment on haemoglobin.

Diagnostic uncertainty may introduce additional bias. The dominant hookworm species present as well as the haemoglobin and diagnostic methods may influence observed impact. Few of the included studies distinguished between the two hookworm species *N. americanus* and *A. duodenale* because of the practical difficulties of differential diagnosis. However, it is suggested that *A. duodenale* causes greater blood loss than *N. americanus* (Pawlowski *et al.* 1991), with data from Zanzibari schoolchildren indicating that *A. duodenale* is associated with an increased risk of anaemia (Albonico *et al.* 1998). Finally, although Hb is routinely assessed as a measure of iron stores, it is insensitive to significant (20–30%) decreases in iron stores from higher Hb levels and is not specific to iron-deficiency anaemia (Zimmermann 2008). Other indicators of iron stores might provide a more sensitive measure of baseline nutritional status and influence the observed impact.

In conclusion, this systematic review confirms the benefits of anthelmintic treatment for improving Hb levels of infected populations but highlights important differences according to the type of benzimidazole drug used and the package of interventions treatment is combined with. This finding highlights the need for continual evaluation of the beneficial effects of deworming on Hb, with randomised evaluations providing the most robust evidence. However, there is also a need for rigorous, long-term evaluation of large-scale control programmes to ensure that they are having maximal benefits for the targeted populations. This would avoid denying individuals the benefits of treatment through randomised evaluations. The results additionally emphasise the public health benefits of combing health interventions and are of particular relevance to efforts implementing an integrated school health package, which may include deworming, iron supplementation, school feeding and malaria control (Bundy *et al.* 2006).

## Footnotes

1 Only two studies among pre-school children (Stoltzfus *et al.* 2000; Brooker *et al.* 1999) were identified and therefore no further analysis among this age group was undertaken.

## Supporting Information

**Table S1.** Baseline characteristics and references of cross-sectional surveys included in the analysis of the impact of hookworm infection on haemoglobin concentration in non-pregnant populations.

**Table S2.** Quality assessment of randomised controlled trials investigating the impact of benzimidazole treatment on haemoglobin
