# Supplementary material for: Impact of hookworm infection and deworming on anaemia in non-pregnant populations: a systematic review
Source: Trop Med Int Health. 2010 Jul;15(7):776–95. doi: 10.1111/j.1365-3156.2010.02542.x (PMC2916221; doi:10.1111/j.1365-3156.2010.02542.x)
Supplement: Supplementary file 1 [file tmi0015-0776-SD1.doc]

**Webtable 1.** Baseline characteristics of cross-sectional surveys included in the analysis of the impact of hookworm infection on haemoglobin concentration in non-pregnant populations

| Study Reference & Author | Setting | Participants and year of study | Diagnostic method | Prevalence of parasites (%) | Prevalence of anaemia (threshold) | Mean intensity (epg)^b^ |
| --- | --- | --- | --- | --- | --- | --- |
| Preschool children | | | | | | |
| [1]  Stolzfus 2000^c^ | Pemba, Zanzibar | Household survey of 614 children aged 5-59 months, 1996 | Kato-Katz | Hw=51.0  Al=39.8  Tt=68.1  Pf=83.7 | 80.4% (<100 g/l)  15.5% (<70 g/l) | All light (1-1,999 epg) infections |
| [2]  Brooker 2007^d^ | Americaninhas, Brazil | Household survey of children aged <5 years, 2004 | Kato-Katz in duplicate, 2 days | Hw= 38.0  Al=47.9  Sm=20.7 | 33.1% (<11g/dl) | 233 |
| School-age children | | | | | | |
| [2]  Brooker 2007^d^ | Americaninhas, Brazil | 332 children in rural schools, aged 5 to 20, 2007 | Kato-Katz in duplicate, 2 days | Hw = 72.9 | 4.5% (<11 g/dL) | 1095 |
| [3]  Stephenson 1989^d^ | Kwale, Kenya | 392 children in rural schools, aged 6 to 17, 1985 | Modified Kato technique | Hw = 93.9  Sh=100  Pf=60.9 | 34.4% (<11 g/dL) | 4493 |
| [4]  Stephenson 1985 | Kwale, Kenya | 250 children in rural schools, aged 6 to 15, 1981 | Kato smear | Hw = 81.2  Pf =42.4  Sh =45.8 | 61.0%(<12 g/dL)^§^ | N/A |
| [5]  Srinivasan 1987 | Chengalpa, South India | Community-based household survey of 346 children aged 5 to 14, 1987 | Kato smear | Hw = 96.2 | 90.0%(<12 g/dL)^§^ | 175 (Geometric) |
| [6] PCD  1998^d^ | Volta region, Ghana | 2125 children in rural schools, aged 8 to 13, 1994 | N/A | Hw = 48.6  Al=7.2  Tt=0.9  Sm=0.0  Sh =13.4 | 15.5% (<11 g/dL) | 121 |
| [7] PCD 1998^d^ | Tanga, Tanzania | 638 children in rural schools, aged 8 to 13, 1996 | N/A | Hw = 63.5  Al=18.2  Tt=9.7  Sm =55.2 | 51.7% (<11 g/dL) | 693 |
| [8]  Stolzfus 1997a&b | Pemba, Zanzibar | 3595 children in rural schools, aged 7 to 16, 1994 | Kato-Katz | Hw = 94.0 | 62.3%(<11 g/dL)^§^ | N/A |
| [9]  Olsen 1998 | Kisumu district, Kenya | Community-based survey of 299 children aged 4-14 years, 1994 | Kato-Katz in duplicate, 2 days | Hw=57.3  Al=27.8  Tt=36.5  Sm=24.4  Pf=6.2 | 17.1% (<11 g/dl) | 47 |
| [10]  Beasley 1999^d^ | Muhenza and Tanga, Tanzania | 1063 children in rural schools, aged 5 to 15, 1994 | Kato-Katz | Hw = 71.7  Al=23.5  Tt=45.4  Pf =45.4  Sh =31.9 | 40.3% (<11 g/dL) | 669 |
| [11]  Miguel & Kremer^d^ | Busia. Kemya | 1041 schoolchildren aged 8 to 20, 1999 | N/A | Hw = 64.2  Al=59.8  Tt=43.0  Sm =66.2 | 16.1% (<11 g/dL) | 561 |
| [12]  Atukorala 1999 | Sri Lanka | 596 female children in rural/urban schools, aged 14 to 18, 1996 | Kato-Katz | Hw = 5.4 | 18.1%(<12 g/dL)^§^ | N/A |
| [13] PCD  unpublished | Eritrea | 1580 schoolchildren aged 4 to 24, 1999 | N/A | Hw = 0.3  Al=0.2  Tt=0.1  Sh=0.0  Sm =2.5 | 7.5% (<11 g/dL) | 0.23 |
| [14] SCF  unpublished | Tousseg, Mali | 390 children in rural schools, aged 5 to 19, 2000 | N/A | Hw = 31.3 | 48.2% (<11 g/dL) | 84 |
| [15] SCF  unpublished | Adjurmani, Burkina Faso | 238 children in rural schools, aged 7 to 21, 2000 | N/A | Hw = 64.7  Al=0  Tt=0.8  Sh=39.5  Sm =0.0 | 21.9% (<11 g/dL) | 190 |
| [16]  Beasley 2002^d^ | Chad | 1001 children in rural schools, aged 6 to 18, 2000 | N/A | Hw = 32.8  Sh=22.5  Sm = 1.0 | 25.1% (<11 g/dL) | 179 |
| [17]  Bhargava 2003^d^ | Pwani, Tanzania | 1030 children in 10 rural schools selected for high prevalence of *S. haematobium*, aged 8 to 14 y, 1997 | Kato-Katz in duplicate | Hw = 70.7  Pf =35.0  Sh=56.4  Al=4.6  Tt=3.6 | 28.7% (<11 g/dL) | 478 |
| [18] Koukounari  2008^d^ | Bondo, Kenya | 1580 children in rural schools, aged 10 to 20,2005 | N/A | Hw = 47.6  Al=23.5  Tt=12.8  Pf =35.0  Sm =14.1 | 13.2% (<11 g/dL) | 215 |
| [19]  Kabatereine 2007^d^ | Uganda | 1380 children in rural schools in 21 districts, aged 2 to 17, 2006 | Kato-Katz | Hw = 5.0  Al=2.9  Tt=4.6  Pf =44.1  Sm =6.4 | 16.7% (<11 g/dL) | 155 |
| [20]  Zhang 2007^d^ | Uganda | 4113 children in rural schools, aged 6 to 15, 2003 | Kato-Katz in duplicate | Hw = 52.1  Al=2.4  Tt=2.4  Sm =44.4 | 34.5% (<11 g/dL) | 296 |
| [21]  Khieu 2006 | Battambang, Cambodia | 168 children aged 6 to 17 years in a rural school, 2004 | Kato-Katz | Hw=54  Al=4  Pf=0 | 24% (<12 g/dl) | 966 |
| [22]  Brooker 2009  unpublished | Tana River, Kenya | 5266 children in rural schools, aged 5 to 20, 2009 | Kato-Katz in duplicate | Hw = 5.0  Pf =3.1  Sm =86.6  Al=2.9  Tt=14.3 | 22.6% (<11 g/dL) | 14.5 |
| Adults | | | | | | |
| [2]  Brooker 2007^d^ | Americaninhas, Brazil | Household survey of adults aged years, 2004 | Kato-Katz in duplicate, 2 days | Hw=72.9  Al=64.2  Sm=43.5 | 30.4% (<12 g/dl females; <13 g/dl males) | 1489 |
| [5]  Srinivasari 1987 | Chengalpa, South India | Community-based household survey of 767 adults aged 15 to 59, 1987 | Kato smear | Hw=80.4 | 83.3% (<12 g/dl females; <13 g/dl males) | 234.4  (Geometric) |
| [8]  Stolzfus 1997a | Pemba, Tanzania | Community-based survey of adult men and non-pregnant women, 1994 | Kato-Katz | N/A | N/A | N/A |
| [9]  Olsen 1998 | Kisumu district, Kenya | Community-based survey of 430 adults aged 15+ years, 1994 | Kato-Katz in duplicate, 2 days | Hw=66.7  Al=7.9  Tt=15.6  Sm=24.9  Pf=25.8 | 18.8% (<11 g/dl for non-pregnant females and <12 g/dl for men) | 74.2 |
| [23]   | Areekil 1979 | | --- | | Bangkok, Thailand | 743 patients aged 15 to 65 years of both sexes with hookworm infection >400 epg admitted to hospital | Stoll’s method | Hw=100 | 43.9% (<12 g/dl) | N/A |
| [24]  Latham 1982 | Kenya | 801 male roadworkers in various area of Kenya, 1978-80 | Ether sedimentation | Hw=39.8  Al=6.4  Tt=32.7  Sm=11.5  Sh=30.5  Pf=18.8 | 23.7% (<13 g/l) | N/A |
| [25]  Nguyen 2006 | Vietnam | National household survey of non-pregnant women, 1995 | Kato-Katz | Hw=36.4  Al=28.2  Tt=58.7 | 39.9% (<12 g/dl) | 270 |

^a^ Restricted to nonmissing hw or hb, exceptions (as presented in paper) marked ^§^

^b^ Arithmetic mean, unless otherwise indicated

^c^ Adjusted for age, prior fever, sex, and malaria parasite density

^d^ Raw data sent by author for analysis

**References for Webtable 1.**

1. Stoltzfus, R.J, Chwaya HM, *et al.* (2000) Malaria, hookworms and recent fever are related to anemia and iron status indicators in 0- to 5-y old Zanzibari children and these relationships change with age. *Journal of Nutrition* **130**: 1724-1733.
2. Brooker, S, Jardim-Botelho A, *et al.* (2007) Age-related changes in hookworm infection, anaemia and iron deficiency in an area of high *Necator americanus* hookworm transmission in south-eastern Brazil. *Transactions of the Royal Society of Tropical Medicine & Hygiene* **101**: 146-154.
3. Stephenson LS, Latham MC, *et al.* (1989) Single dose metrifonate or praziquantel treatment in Kenyan children. II. Effects on growth in relation to *Schistosoma haematobium* and hookworm egg counts. *American Journal of Tropical Medicine and Hygiene* **41**:445-453. Raw data contributed by author.
4. Stephenson LS, Latham MC, *et al.* (1985) Relationships of *Schistosoma hematobium*, hookworm and malarial infections and metrifonate treatment to hemoglobin level in Kenyan school children. *American Journal of Tropical Medicine and Hygiene* **34**: 519-528.
5. Srinivasan V, Radhakrishna S, *et al.* (1987) Hookworm infection in a rural community in South India and its association with haemoglobin levels. *Transactions of the Royal Society of Tropical Medicine and Hygiene* **81**: 973-977.
6. Partnership for Child Development (1998) The health and nutritional status of schoolchildren in Africa: evidence from school-based health programmes in Ghana and Tanzania. *Transactions of the Royal Society of Tropical Medicine and Hygiene* **92**: 254-450. Raw data contributed by author (Ghana).
7. Partnership for Child Development (1998) The health and nutritional status of schoolchildren in Africa: evidence from school-based health programmes in Ghana and Tanzania. *Transactions of the Royal Society of Tropical Medicine and* Hygiene **92**: 254-450. Raw data contributed by author (Tanzania).
8. Stoltzfus RJ, Chwaya HM, *et al.* (1997a) Epidemiology of iron deficiency anemia in Zanzibari schoolchildren: The importance of hookworms. *American Journal of Clinical Nutrition* **65**: 153-159.
9. Stoltzfus RJ, Dreyfuss ML, *et al.* (1997b) Hookworm control as a strategy to prevent iron deficiency. *Nutrition Reviews* **55**: 223-232.
10. Olsen A, Magnussen P, *et al.* (1998) The contribution of hookworm and other parasitic infections to haemoglobin and iron status among children and adults in western Kenya. *Transactions of the Royal Society of Tropical Medicine and Hygiene* **92**: 643-649.
11. Beasley NMR, Tomkins AM, *et al.* (1999) The impact of population level deworming on the haemoglobin levels of schoolchildren in Tanga, Tanzania. *Tropical Medicine and International Hleath* **4**: 744-750. Raw data contributed by author.
12. Miguel EA and Kremer M (2004) Worms: Identifying impacts of eduction and health in the presence of treatment externalities. *Econometrica* **72**:159-217. Raw data contributed by author.
13. Atukorala, T. M. S. and P. Lanerolle (1999) Soil-transmitted helminthic infection and its effect on nutritional status of adolescent schoolgirls of low socioeconomic status in Sri Lanka. *Journal of Tropical Pediatrics* **45**: 18-22.
14. Partnership for Child Development (1999), unpublished raw data, Eritrea
15. Save the Children Fund (2000), unpublished raw data, Mali
16. Save the Children Fund (2000), unpublished raw data, Burkina Faso
17. Beasley M, Brooker S, Ndinaromtan M, *et al.*(2002) First nationwide survey of the health of schoolchildren in Chad. *Tropical Medicine and International Health* **7**:625-30. Raw data contributed by author.
18. Bhargava, A., M. Jukes, *et al.* (2003) Anthelmintic treatment improves the hemoglobin and serum ferritin concentrations of Tanzanian schoolchildren. *Food & Nutrition Bulletin* **24**: 332-342. Raw data contributed by author.
19. Koukounari A, Estambale BB, *et al.* (2008) Relationships between anaemia and parasitic infections in Kenyan schoolchildren: a Bayesian hierarchical modelling approach. *International Journal of Parasitology* **38**: 1663-71. Raw data contributed by author.
20. Kabatereine, N. B., S. Brooker, *et al.* (2007) Impact of a national helminth control programme on infection and morbidity in Ugandan schoolchildren. *Bulletin of the World Health Organization* **85**: 91-99. Raw data contributed by author.
21. Zhang Y, Koukounari A, *et al.* (2007) Parasitological impact of a 2-year preventive chemotherapy on schistosomiasis and soil-transmitted helminthiasis in Uganda *BMC Medicine* **3**: 5-27. Raw data contributed by author.
22. Khieu V, Odermatt P, *et al.* (2006) Anaemia in a school of rural Cambodia: detection, prevalence, and links with intestinal worms and malnutrition. *Bulletin de la Societe de Pathologie Exotique* **99**: 115-118.
23. Brooker S (2009) unpublished raw data, Kenya
24. Areekul S (1979) The relationship between anaemia and hookworm infection. *Journal of The Medical Association of Thailand* **62**: 379-382.
25. Latham, MC, Stephenson LS, *et al.* (1982) A comparative study of the nutritional status, parasitic infections and health of male roadworkers in four areas of Kenya. *Transactions of the Royal Society of Tropical Medicine & Hygiene* **76**: 734-740.
26. Nguyen PH, Nguyen KC, *et al.* (2006) Risk factors for anemia in Vietnam. *Southeast Asian Journal of Tropical Medicine & Public Health* **37**: 1213-1223.

**Webtable 2**. Quality assessment of randomised controlled trials investigating the impact of benzimidazole treatment on haemoglobin

| Study | Adequate sequence generation | Allocation concealment | Placebo | Blinding | Hb diagnostic test | Parasitological method | Time to follow-up (duration, treatment schedule) | Loss to follow-up |
| --- | --- | --- | --- | --- | --- | --- | --- | --- |
| Kenya  (Stephenson 1990) | Not described | Unclear | Yes | Unclear | Duplicate | Kato^a^ | 7 weeks | 8.3%. Reasons described. |
| Kenya, 1989 (Stephenson 1993) | Not described | Unclear | Yes | Unclear | Duplicate | Kato^a^ | 4 months | 12%. No further description |
| Kenya, 1990  (Adams 1994) | Not described | Not used | Yes | Unclear | Duplicate | Kato^a^ | 9 weeks | 1.8%. No further description |
| Tanzania, 1994 (Stoltzfus 1998) | Not described | Unclear | No | None | Unclear | Kato-Katz^a^ | 4 months (1 year, treated thrice yearly) | 16%. Referred to publication with further details (dropouts more likely to be male and not stunted) |
| North India, 1995 (Awasthi 2000)^l^ | Odd or non-zero serial numbers to placebo | Unclear | Yes | Single blind | Unclear | Direct smear^a^ | 6 months (2 yrs, treated every 6 mo.) | 2%. No further description |
| Benin,  (Dossa 2001) | Not described | Unclear | Yes | Double blind | Unclear | Kato^a^ | 2 months (3 months, treated at 0 and 1 mo.) | 1.1% Dropouts did not differ in any background characteristics |
| Bangladesh,  (Gilgen 2001) | Random Numbers | Unclear | Yes | Unclear | Unclear | Formalin-ether concentration^a^ | 12 weeks (24 weeks, treated at 0 and 12 wk.) | 7.4%. Not addressed in text. |
| Tanzania  (Stolzfus 2004) | Not described | Unclear | Yes | Double blind | Unclear | Kato-Katz^a^ | 3 months (1 yr, treated every 3 mo.) | 14%. Characteristics of dropouts described |
| Viet Nam, 2005  (Le Huong 2007) | Not described | Unclear | Yes | Double blind | Unclear | Kato-Katz (10% in duplicate) | 3 months (6 months, treated at 0 and 3 mo.) | 13%. Not addressed in text. |
| Viet Nam, 2007  (Nga 2009) | Computer generated | Yes | Yes | Double blind | 10% in duplicate | Kato-Katz^a^ | 4 months | 8.6%. Dropouts did not differ in any background characteristics |
| Tanzania, 1994 (Beasley 1999) | Random Numbers | Unclear | Yes | Single blind | Unclear | Kato-Katz (single) | 15 weeks | 30%. Characteristics of dropouts described |
| South Africa , 1996 (Taylor 2001) | Not described | Unclear | Yes | Double blind | Unclear | Formol-ether concentration^a^ | 6 months (1 year, treated every 6 mo.) | 36%. Not addressed in text. |
| Kenya, 2003  (Friis 2003) | Not described | Unclear | Yes | Double blind | Unclear | Kato-Katz (duplicate) | 8 months | 23%. Characteristics of dropouts described |
| Côte d’Ivoire, 2007 (Rohner 2010) | Not described | Yes | Yes | Double blind | Unclear | Kato-Katz (duplicate) | 3 months (6 months, treated at 0 and 3 mo.) | 6.3%. Not addressed in text. |

^a^ Number of smears unclear, assume to be single
